# Cathelicidin-Related Antimicrobial Peptide Regulates CD73 Expression in Mouse Th17 Cells via p38

**DOI:** 10.3390/cells9061561

**Published:** 2020-06-26

**Authors:** Jeonghyun Lee, Kyong-Oh Shin, Yesol Kim, Jaewon Cho, Hyung W. Lim, Sung-Il Yoon, Geun-Shik Lee, Hyun-Jeong Ko, Pyeung-Hyeun Kim, Yoshikazu Uchida, Kyungho Park, Seung Goo Kang

**Affiliations:** 1 Division of Biomedical Convergence, College of Biomedical Science, Kangwon National University, Chuncheon 24341, Korea; jhleee@kangwon.ac.kr (J.L.); iamfine@kangwon.ac.kr (Y.K.); sungil@kangwon.ac.kr (S.-I.Y.); 2 Department of Food Science and Nutrition, and Convergence Program of Material Science for Medicine and Pharmaceutics, Hallym University, Chuncheon 24252, Korea; 0194768809@hanmail.net; 3 College of Pharmacy, Kangwon National University, Chuncheon 24341, Korea; jwcho@kangwon.ac.kr (J.C.); hjko@kangwon.ac.kr (H.-J.K.); 4 Gladstone Institute of Virology and Immunology, Gladstone Institute of Neurological Disease, School of Medicine, Department of Neurology, University of California, San Francisco, CA 94158, USA; hyungwook.lim@gmail.com; 5 Institute of Bioscience & Biotechnology, Kangwon National University, Chuncheon 24341, Korea; leegeun@kangwon.ac.kr (G.-S.L.); phkim@kangwon.ac.kr (P.-H.K.); 6 College of Veterinary Medicine, Kangwon National University, Chuncheon 24341, Korea; 7 Department of Molecular Bioscience, School of Biomedical Science, Kangwon National University, Chuncheon 24341, Korea; 8 Department of Dermatology, School of Medicine, University of California, San Francisco and Northern California Institute for Research and Education, Veterans Affairs Medical Center, San Francisco, CA 94212, USA; Yoshikazu.Uchida@ucsf.edu

**Keywords:** CRAMP, antimicrobial peptide, CD73, adenosine, Th17 cells, TGF-beta, p38

## Abstract

The effector function of tumor-infiltrated CD4^+^ T cells is readily suppressed by many types of immune regulators in the tumor microenvironment, which is one of the major mechanisms of immune tolerance against cancer. Cathelicidin-related antimicrobial peptide (CRAMP), the mouse analog of LL-37 peptide in humans, is a cationic antimicrobial peptide belonging to the cathelicidin family; however, its secretion by cancer cells and role in the tumor microenvironment (TME) remain unclear. In this study, we explored the possibility of an interaction between effector CD4^+^ T cells and CRAMP using in vitro-generated mouse Th17 cells. We found that CRAMP stimulates Th17 cells to express the ectonucleotidase CD73, while simultaneously inducing cell death. This finding suggested that CD73-expressing Th17 cells may function as immune suppressor cells instead of effector cells. In addition, treatment of pharmacological inhibitors of the transforming growth factor-beta (TGF-β) signaling pathway showed that induction of CD73 expression is mediated by the p38 signaling pathway. Overall, our findings suggest that tumor-derived LL-37 likely functions as an immune suppressor that induces immune tolerance against tumors through shaping effector Th17 cells into suppressor Th17 cells, suggesting a new intervention target to improve cancer immunotherapy.

## 1. Introduction

CD4^+^ T cells and tumors interact in the tumor microenvironment (TME), and this interaction is a strong determinant of tumor growth or eradication [1,2]. This paradoxical outcome is related to the complexity of the TME and the flexibility of T cell functions [3]. Tumors generate a suppressive environment for CD4^+^ T cells by expressing and secreting various inhibitory ligands and suppressive molecules such as metabolites and cytokines [4,5]. Modulating the suppressive TME to enhance T cell immunity via blockade of these inhibitory ligands has shown great success in the treatment of certain cancer types, including antibodies targeting programmed death (PD)-1 and its ligand [6]. However, substantial efforts are still needed to define the immune modulatory molecules in the TME and elucidate the underlying mechanisms by which T cell immunity is suppressed.

Extracellular adenosine is a potent immune-suppressive molecule that supports tumor growth in the TME, and its concentration in tumors is much higher than that in adjacent normal tissues [7]. Among the four adenosine receptors (A1, A2a, A2b, and A3), myeloid cells and lymphocytes express A2a and A2b, and extracellular adenosine suppresses their immune functions [8,9]. Adenosine is generated by extracellular ATP via the ectonucleotidases CD39 and CD73, which are expressed on the cell surface [10]. CD73, encoded by *NT5E*, is a rate-limiting factor for adenosine accumulation in the TME as it is the only enzyme capable of breaking down AMP into adenosine [8,11]. CD39 converts ATP to AMP; however, AMP in the TME is also generated by the action of other enzymes expressed on the surface of tumor or regulatory cells, including CD38 and CD203a [12]. CD73 is widely expressed on tumor cells and inhibits antitumor T cell-mediated immunity [13], and is also expressed on tumor-infiltrated immune cells, contributing to the generation of the immunosuppressive TME. Interestingly, deficiency of CD73 in either hematopoietic or non-hematopoietic cells was reported to be sufficient to invigorate antitumor immunity [14,15], suggesting that the overall expression level of functional CD73 from both tumor cells and immune cells in the TME is a key factor determining the consequence of tumor growth or eradication.

Transforming growth factor (TGF)-β is one of the most well-described CD73 inducer cytokines that is enriched in the TME and is an essential cytokine for the generation and functions of regulatory T cells (Tregs) [16,17]. Indeed, Tregs in tumor tissues express a high level of CD73, which is a critical factor for their suppressive function in tumors [15]. Accordingly, CD73-depleted Tregs cannot suppress the antitumor immunity response, allowing the host immune system to mount a defense against tumors [15]. A recent study showed that in vitro-generated Th17 cells expressing interleukin (IL)-6 and TGF-β also express CD73 in a signal transducer and activator of transcription 3 (STAT3) and growth factor independence 1 (Gfi-1)-dependent manner. IL-6 directly induces STAT3, whereas TGF-β suppresses the expression of the inhibitory transcription factor Gfi-1 via the p38 signaling pathway [18]. Moreover, Th17 cells induced in the absence of TGF-β do not express CD73, and adoptive transfer of these cells to tumor-bearing mice effectively suppressed tumor growth, whereas transfer of the TGF-β-induced Th17 cells had a protumor effect [18]. These results suggest that the immunosuppressive environment is created by not only the presence of suppressive cells such as Tregs but also by the conversion of effector T cells to a tumor-permissive cell type via interactions with immune modulators.

Cathelicidins, including human cathelicidin antimicrobial peptide (CAMP) and the mouse analog cathelicidin-related antimicrobial peptide (CRAMP), are antimicrobial peptides produced by various cell types, including epithelial cells and immune cells [19]. Cathelicidin is then further processed by proteases to the bioactive form LL-37 (with 37 amino acid residues) [20]. LL-37 exhibits diverse functions beyond its key role in antimicrobial activities, including induction of cytokine secretion and chemotaxis [21]. In addition, several types of tumors, such as breast cancer, pancreatic tumor, and skin squamous cell carcinoma, contain higher concentrations of LL-37 than normal tissues, which plays a direct role in tumor growth [22,23]. However, the interaction between LL-37 and the tumor can also be indirect, given the expression of its known receptors such as FPRL-1, P2x7, epidermal growth factor receptor (EGFR), and GAPDH on both tumor cells and various types of immune cells [23,24,25,26], implicating the immunomodulatory roles of LL-37 via a direct interaction with immune cells. Indeed, a recent study demonstrated that CRAMP produced from pancreatic β-cells generates regulatory macrophages via EGFR expressed on macrophages in mice [27]. Since these receptors are also expressed on T cells, it is highly possible that CRAMP acts directly on T cells to regulate their functions.

Therefore, we hypothesized that CRAMP is a potent factor responsible for suppressing immunity in the TME. To test this hypothesis, we explored the effects of CRAMP on the apoptosis, differentiation, and functions of mouse CD4^+^ T cells using an in vitro culture system. Elucidation of this mechanism may reveal important features of CRAMP as an immune regulator in certain circumstances, such as in the TME, highlighting a new target for cancer immunotherapy.

## 2. Materials and Methods

### 2.1. Animals

Eight to ten-week old female C57BL/6J mice (The Jackson Laboratory, Bar Harbor, ME, USA) were used in all experiments. All mice were maintained at Kangwon National University Animal Laboratory Center and were used in accordance with the guidelines from the Animal Care and Use Committee of Kangwon National University. All of the animal related experiments were done under the approval of Institutional Animal Care and Use Committee (IACUC, project identification code: KW-160429-1).

### 2.2. Naïve CD73^−^CD4^+^ T Cell Isolation

Splenocytes were collected from the C57BL/6J mice and red blood cells were lysed with ACK lysing buffer (Gibco, now Thermo Fisher Scientific, Waltham, MA, USA), followed by isolation using the naïve CD4^+^ T cell isolation kit (Miltenyi Biotec, Bergisch Gladbach, Germany) with some modifications. In brief, the splenocytes were stained with a biotinylated non-CD4^+^ T cell antibody cocktail provided in the naïve CD4^+^ T cell isolation kit together with phycoerythrin (PE)-conjugated anti-CD8 (clone 53-6.7), anti-CD19 (clone 6D5), anti-CD25 (clone PC61), anti-CD69 (clone H1.2F3), anti-CD44 (clone IM7), and anti-CD73 (clone TY/11.8) antibodies for 20 min at 4 °C. The stained splenocytes were washed with MACS buffer (0.5% bovine serum albumin (BSA), 2 mM ethylenediaminetetraacetic acid (EDTA) in Dulbecco’s phosphate-buffered saline (DPBS)) and incubated with anti-biotin beads (Miltenyi Biotec) and anti-PE beads (Miltenyi Biotec) for 20 min at 4 °C. Naïve CD73^−^CD4^+^ T cells were negatively isolated using an LS column (Miltenyi Biotec), and the isolated CD4^+^CD62L^hi^ cells (>95%) were used for in vitro T cell culture. The monoclonal antibodies were purchased from BD Biosciences (San Jose, CA, USA), eBioscience (now Thermo Fisher Scientific, Waltham, MA, USA) or Biolegend (San Diego, CA, USA).

### 2.3. In Vitro T Cell Culture System

Naïve CD4^+^ T cells (5 × 10^4^) were cultured in an anti-CD3 (5 μg/mL, Biolegend, clone 145-2C11) pre-coated 96-well plate with anti-CD28 (2 μg/mL, Biolegend, clone 37.51) for 3 days (Treg, Th1, Th2 cells) or 5 days (Th17 cells), and the following cytokines were added according to specific cell differentiation conditions: hTGF-β (2 ng/mL, Peprotech, Rocky Hill, CT, USA) and hIL-2 (50 U/mL, Peprotech) for Treg cells; mIL-12 (2 ng/mL, Peprotech), α-IL-4 (10 μg/mL, BioXcell, Lebanon, PA, USA), and hIL-2 (50 U/mL, Peprotech) for Th1 cells; IL-4 (2 ng/mL, Peprotech), α-interferon (IFN)-γ (10 μg/mL, BioXcell), and hIL-2 (50 U/mL, Peprotech) for Th2 cells; hTGF-β (2 ng/mL, Peprotech), mIL-1β (10 ng/mL, Peprotech), mIL-21 (10 ng/mL, Peprotech), mIL-23 (10 ng/mL, R&D, Minneapolis, MN, USA), mIL-6 (20 ng/mL, Peprotech), α-IL-4 (10 μg/mL, BioXcell), and α-IFN-γ (10 μg/mL, BioXcell) for Th17 cells. All cells were cultured in complete GlutaMAX RPMI-1640 medium (Gibco, now Thermo Fisher Scientific) supplemented with 10% fetal bovine serum (Gemini Bio, West Sacramento, CA, USA), 1% penicillin/streptomycin (Gibco), 5μg/mL Gentamicin (Gibco), 50 μM 2-mercaptoethanol (Gibco), and 10 mM HEPES (Gibco).

To assess the effects of CRAMP stimulation on effector cells, in vitro-differentiated or Th17 cells (1 × 10^5^) or Tregs (1 × 10^5^) were cultured for 2 more days in an anti-CD3 (5 μg/mL) pre-coated or non-coated 96-well plate, respectively, with anti-CD28 (2 μg/mL) and hTGF-β (0.1 ng/mL) in the presence or absence of CRAMP (10 μM, Cat# AS-61305, Anaspec, Fremont, CA, USA). The cells were also treated with SB431542 (10 μM, Cayman Chemical, Ann Arbor, MI, USA) or SB203580 (30 μM, Merck, St. Louis, MO, USA) during this additional 2 day culture period.

For the cell death assay, isolated naïve CD73^−^CD4^+^ T cells (5 × 10^4^) were activated in an anti-CD3 (5 μg/mL) pre-coated 96-well plate with anti-CD28 (2 μg/mL) and hIL-2 (50 U/mL) for 3 days in the presence of various concentrations of CRAMP.

### 2.4. Flow Cytometry Analysis

Intracellular expression of FoxP3, IFN-γ, IL-4, and IL-17A was measured by flow cytometry. The cultured cells were harvested and stained for cell surface markers in MACS buffer containing Live/Dead fixable Near IR Dead cell stain dye (APC/cy7, Thermo Fisher) for 20 min at 37 °C and incubated in complete RPMI-1640 medium containing a cell stimulation cocktail (Invitrogen) for 3 h at 37 °C. The cells were then fixed and permeabilized for 1 h at 4 °C with Fixation/Permeabilization Buffer (eBioscience, now Thermo Fisher Scientific). Dye-conjugated antibodies for cytokines and surface markers were diluted in 1× permeabilization buffer (eBioscience) and incubated with the cells at 4 °C for 30 min. After washing with 1× permeabilization buffer, the cells were subjected for FACS analysis. The following antibodies were used for surface staining: anti-CD4 (Percp/cy5.5, clone GK1.5), anti-CD73 (PE, clone TY/11.8) and antithy1.1 (Pacific Blue, clone OX-7) for virus infected Th17 cells. The following antibodies were used for intracellular staining: anti-FoxP3 (PE/cy7, clone FJK-16s), anti-IFN-γ (APC, clone XMG1.2), anti-IL-4 (BV421, clone 11B11), and anti-IL-17A (AF647, clone TC11-18H10.1). All antibodies were purchased from Biolegend (San Diego, CA, USA) and eBioscience (now Thermo Fisher Scientific).

### 2.5. Reverse Transcription-Polymerase Chain Reaction (RT-PCR)

Total RNA was extracted using TRIzol Reagent (Invitrogen, Carlsbad, CA, USA) and subjected to cDNA synthesis using the iScript gDNA clear cDNA synthesis kit (Bio-Rad, Hercules, CA, USA) according to the manufacturer’s instructions. PCR was then performed for the target genes using the cDNA as the template with Maxima SYBR Green/ROX qPCR Master Mix (Thermo Fisher Scientific) on an Applied Biosystems 7500 system (Thermo Fisher Scientific) and normalized by the expression level of the cyclophilin A gene. Quantitative PCR was performed over 40 cycles of 95 °C (60 s) for DNA denaturation and 60 °C (60 s) for primer annealing and extension. The cycle threshold (C_T_) value for each gene was then used for calculation of the relative expression level as 1/2^CT^, and divided by the expression level (1/2^CT^) of cyclophilin A for normalization. The sequences of the primers were *Nt5e* Forward: 5′-GGAAACCTGATCTGTGATGC-3′, *Nt5e* Reverse: 5′-CTTCAGGGTGGACCCTTTTA-3′; *Gfi-1* Forward: 5′-AGGCGAGTCGAAAATGGAG-3′, *Gfi-1* Reverse: 5′-AGAGAGCGGCACAGTGACTT-3′; cyclophilin A Forward: 5′-GGCCGATGACGAGCCC-3′ and cyclophilin A Reverse: 5′-TGTCTTTGGAACTTTGTCTGCAA-3′.

### 2.6. Adenosine Quantification

Th17 cells (1 × 10^5^) were incubated in Hank’s balanced salt solution with AMP (1 mM) for 1 h, and the culture supernatant was collected. The quantitative analysis of adenosine and AMP was performed by LC-ESI-MS/MS (API 3200 QTRAP mass, AB/SCIEX, Toronto, Canada) as described previously with minor modifications. Prior to the extraction of adenosine, deproteinization from the cell culture supernatants (0.1 mL) was conducted by adding acetonitrile (0.4 mL), including 100 pmol of internal standards (Adenosine-^15^N_5_ 5′-monophosphate, Adenosine-^15^N_5_). Adenosine and AMP were separated by reverse-phase high-performance liquid chromatography (HPLC) (NANOSPACE SI-2 HPLC equipped with HTS autosampler Z, Shiseido, Tokyo, Japan) using a KINETEX C18 column (2.1 × 50 mm, ID: 2.6 μm; Phenomenex, St. Louis, MO, USA). Mobile phase A was water with 0.1% formic acid, and mobile phase B was 50% acetonitrile with 0.1% formic acid. The initial gradient of the mobile phase was maintained at 95% phase A for 3 min, and the linear gradient to 100% phase B was achieved in 4 min and maintained for 2.5 min, followed by a switch back to 95% solvent A in 1 min that was further maintained for additional 5 min. The extracts were analyzed by LC-ESI-MS/MS using the selective ion monitoring mode. The tandem mass spectrometry (MS/MS) transitions (*m*/*z*) of the analytes were 348.1→136.1 for AMP, 353.1→141.1 for Adenosine-^15^N_5_ 5′-monophosphate, 268.1→136.1 for adenosine, and 273.1→141.1 for a denosine-^15^N_5_. Data were acquired using Analyst 1.5.1 software (Applied Biosystems, Foster City, CA, USA). The levels of adenosine and AMP were quantitated using calibration curves with various concentrations of analytes and internal standard ratios.

### 2.7. Cell Death Assay

The CRAMP-stimulated activated Treg or Th17 cells were harvested and stained with the Annexin V/PI staining Kit (eBioscience) according to the manufacturer’s instructions. In brief, the cells were incubated with Annexin V-APC, diluted 1:20 in 1× binding buffer for 20 min at room temperature and then, with propidium iodide (PI), diluted 1:40 in 1× binding buffer for 5 min at room temperature. One microliter of antibodies was used for each sample. PI and anti-CD4 (BV421, clone GK1.5) antibodies were stained together.

### 2.8. Plasmids and Retroviral Gene Transduction

*Gfi-1* (Gfi-1 NGFR, Addgene plasmid #44630) template DNA (Addgene, Watertown, MA, USA) was amplified by PCR using specific primers (Forward 5′-ATGCCTCGAGATGCCGCGCTCATTCCTGGT-3′ and Reverse 5′-ATGCACGCGTTCATTTGAGTCCATGCTGAGT-3′) and inserted into a Thy-1.1-expressing retroviral vector (Addgene plasmid #17442). S-Eco packing cells were transfected by JetPrime transfection kit (Polyplus-transfection SA, Illkirch-Graffenstaden, Alsace, France) and retroviral supernatants were collected 48 h after transfection. For retroviral infection, 1 day-cultured Th17 cells were subjected to spin-infection with the retroviral supernatant supplemented with 8 µg/mL polybrene (Merck Millipore, Burlington, MA, USA) at 1500× *g* for 90 min at 30 °C, followed by 4 more days of culture in the Th17 differentiation condition. The retrovirus-infected Th17 cells were cultured 2 more days as described above and then, subjected for CD73 staining.

### 2.9. Statistical Analysis

All data presented as bar graphs represent mean ± SEM. P-values were determined using a two-tailed Student *t*-test (for two-group comparisons) or one-way analysis of variance with the post hoc Tukey test (for multiple-groups comparisons). The “*n*” values in the figures and legends represent the number of independent experiments performed. GraphPad Prism 7 software was used for the statistical analysis.

## 3. Results

### 3.1. CRAMP Induces the Apoptosis of CD4^+^ T Cells

**Figure 1:**
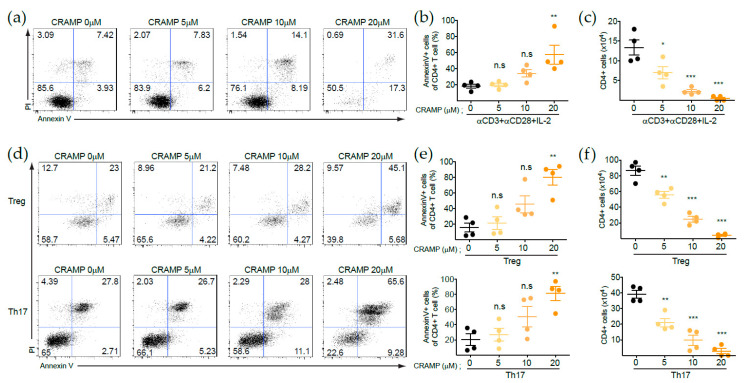
CRAMP induces the apoptosis of CD4^+^ T cells. (**a**–**c**) Naïve CD4+ T cells were activated with anti-CD3/CD28 in the presence of various concentrations of CRAMP for three days and subjected to Annexin V/PI staining. Flow cytometry analysis (**a**), frequency of apoptotic cells (Annexin V+ cells) (**b**), and absolute number of live cells (**c**) are indicated (*n* = 4). (**d**–**f**) Naïve CD4+ T cells were differentiated into Tregs and Th17 cells in vitro in the presence of various concentrations of CRAMP for three or five days. Differentiated Tregs and Th17 cells were then subjected for Annexin V/PI staining and analyzed by flow cytometry (**d**). The frequency (**e**) and absolute number (**f**) of live cells are indicated (*n* = 4). * *p* < 0.05, ** *p* < 0.01, *** *p* < 0.001, n.s—not significant (one-way ANOVA with post hoc Tukey test).

LL-37 has different functions depending on its concentration and acts as an antimicrobial peptide via disrupting the cell membrane when present at high concentrations [21,28]. Therefore, it is plausible that CRAMP has a direct effect on T cell viability as one of the main immune-regulatory modes in the TME [29,30]. To test this possibility, we first explored the potential pro-apoptotic role of CRAMP during T cell activation. Naïve T cells isolated from C57BL/6J mice were activated with anti-CD3/CD28 antibody in the presence of various concentrations of CRAMP. Previous studies showed that the concentration of LL-37 (the human analog of CRAMP) in serum was about 0.26 μM, which could be elevated up to 7 μM in the bronchoalveolar lavage fluid of the lung in cystic fibrosis patients. In addition, immune modulatory functions of LL-37 were observed in the range of 2.2–11.1 μM [31,32]. Therefore, we considered that ~10 μM of CRAMP is the highest biologically meaningful concentration for evaluation in this study. We further examined the effects of a higher concentration of CRAMP (20 μM) because it is plausible that concentrations above 10 μM could accumulate via secretion of CRAMP from the tumor cell itself and/or tumor-infiltrated macrophages [33]. Indeed, most of the activated T cells were found to undergo cell death after treatment with 20 μM of CRAMP and apoptosis was also induced with treatment of the physiological concentration (~10 μM) of CRAMP (Figure 1a,b) [34]. Therefore, the total number of activated CD4^+^ T cells was inversely correlated with the CRAMP concentration (Figure 1c).

Since CRAMP can exert effects on differentiated effector T cells in certain environments such as the TME, we also evaluated whether apoptosis occurred in effector T cells via CRAMP. In vitro-differentiated Tregs and Th17 cells were stimulated with anti-CD3/CD28 along with CRAMP, and both types of effector T cells were also found to undergo cell death under a high concentration of CRAMP (Figure 1d–f). These results indicated that CRAMP directly acts on T cells to induce apoptosis, suggesting that it is one of the key factors responsible for cell death-mediated immune regulation in certain environments, including the TME.

### 3.2. CRAMP Induces CD73 Expression on CD4^+^ T Cells

**Figure 2:**
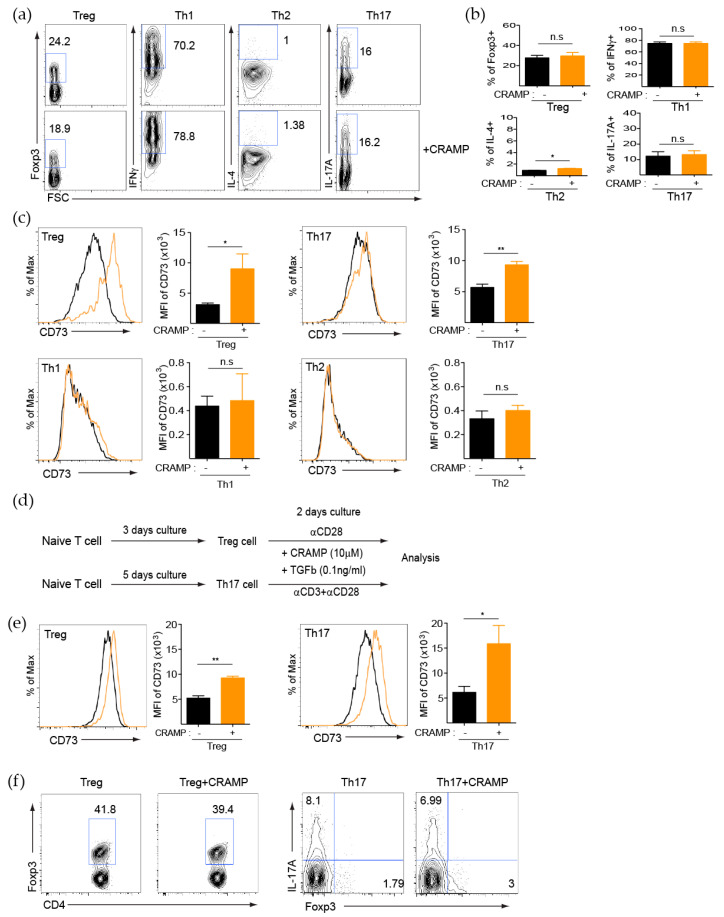
CRAMP induces CD73 expression on CD4^+^ T cells. (**a**–**c**) Naïve CD4^+^ T cells were cultured under each differentiation condition with or without CRAMP (10 μM) for three days (Th1, Th2, and Treg cells) or five days (Th17 cells). Each in vitro-differentiated subset was stained for their representative markers. Flow cytometry analysis (**a**) and quantification of subsets (**b**) are indicated (*n* = 4). (**c**) Flow cytometry analysis of CD73 expression for each subset; the bar graph indicates the mean fluorescent intensity (MFI) value of CD73 (*n* = 4). (**d**–**f**) In vitro-differentiated Treg or Th17 cells were stimulated for two days more with or without CRAMP. (**d**) Experimental scheme of Treg or Th17 cells stimulation for two days with CRAMP. (**e**) Flow cytometry analysis of CD73 expression; the bar graph indicates the MFI value of CD73 (*n* = 3). (**f**) Flow cytometry analysis of cellular FoxP3 expression level. A representative result from three independent experiments is shown. * *p* < 0.05, ** *p* < 0.01, n.s—not significant (Student’s *t*-test).

Since the modulation of effector T cell generation is one of the key modes of immune regulation, we next examined whether CRAMP regulates the generation of diverse subsets of CD4^+^ T cells, including Th1, Th2, Th17, and Tregs. However, CRAMP did not alter the generation of each subset of CD4^+^ T cells compared with those of the untreated control group (Figure 2a,b). We further explored the possibility that CRAMP regulated the expression of functional molecules on CD4^+^ T cells, as another important mode of immune regulation. CD73 is one of the functional molecules regulated by p38 via TGF-β, and CRAMP was reported to stimulate the p38 signaling pathway in immune cells [18,35]. Therefore, we reasoned that CRAMP may regulate CD73 expression on effector T cells such as Tregs and Th17 cells, which depend on TGF-β signaling for their generation. Indeed, in vitro-differentiated Tregs and Th17 cells expressed significantly more CD73 on the cell surface after CRAMP treatment, whereas Th1 and Th2 cells did not (Figure 2c).

Since CRAMP is highly expressed in several tissues, including the skin, intestine, and certain types of tumors, it is possible that it had already affected differentiated effector T cells. Therefore, we examined whether CRAMP also stimulates effector T cells to upregulate CD73. To test this possibility, Tregs and Th17 cells were generated from naïve CD4^+^ T cells in vitro, and then, cultured for two more days in the presence or absence of CRAMP (Figure 2d). Higher expression levels of CD73 were observed in response to CRAMP in the presence of TGF-β and anti-CD3 stimulation, and this effect was more prominent in Th17 cells than in Tregs (Figure 2e). Next, we examined whether CRAMP modulates forkhead box protein 3 (FoxP3) expression in Treg or Th17 cells because Th17 cells have a plastic phenotype and can differentiate into IL-17A^+^ FoxP3^+^ or IL-17A-FoxP3^+^ suppressive cells in the TME [36]. However, CRAMP did not alter the expression of FoxP3 in Tregs or Th17 cells (Figure 2f). These results suggested that CRAMP promotes the conversion of tissue-infiltrated effector T cells to suppressive T cells through the upregulation of CD73 without altering FoxP3 expression.

### 3.3. CRAMP-Induced CD73 Is Functional

**Figure 3:**
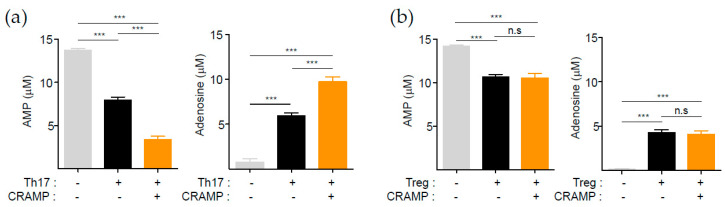
CRAMP-induced CD73 is functional. (**a**,**b**) AMP and adenosine quantification assay. In vitro-differentiated Treg and Th17 cells were cultured for 2 or more days as indicated as Figure 2d. Tregs (1 × 10^5^) or Th17 cells (1 × 10^5^) were incubated with 1 mM AMP for 1 h. AMP and adenosine were quantified by LC-ESI-MS/MS. Treg cells (*n* = 3), Th17 cells (*n* = 4). *** *p* < 0.001 and n.s—not significant (one-way ANOVA with post hoc Tukey test).

CD73 is an ectonucleotidase that converts AMP to adenosine, which is a well-known potent immune suppressor [8]. Since the in vitro-generated Th17 cells, differentiated in the presence of TGF-β, already expressed a high level of CD73 (Figure 2c), we questioned whether further induction of CD73 by CRAMP would be physiologically meaningful. We therefore compared the adenosine-generating ability between control Th17 cells and CRAMP-stimulated Th17 cells after culture with exogenous AMP for 2 h. Interestingly, CRAMP-treated Th17 cells substantially degraded AMP, leading to the generation of a greater amount of adenosine compared to that produced by untreated control Th17 cells (Figure 3a). However, no augmented enzyme activity was observed in Tregs under the same condition (Figure 3b). These results suggest that the CD73 expression level per cell is positively correlated with the ability of adenosine generation, highlighting CRAMP as a potential factor responsible for generating anti-inflammatory Th17 cells.

### 3.4. p38 Is Responsible for the CRAMP-Mediated Induction of CD73

**Figure 4:**
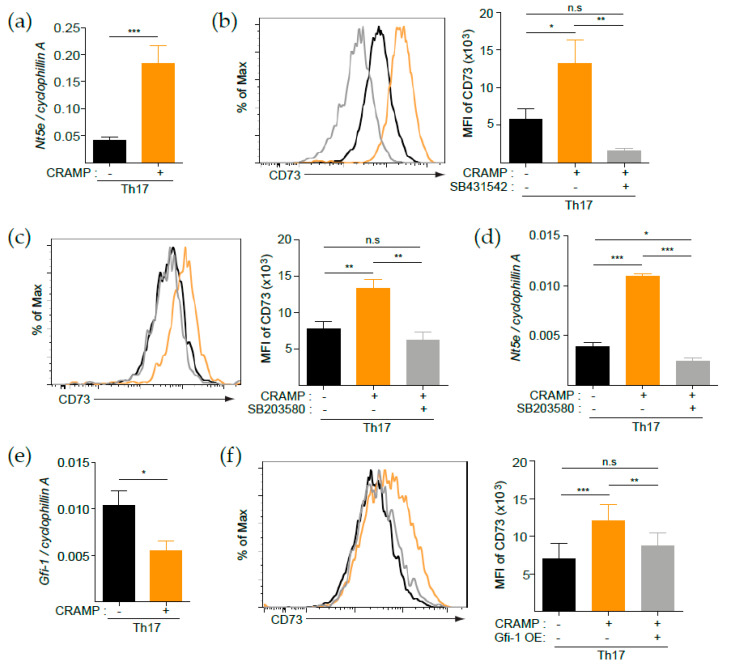
p38 is responsible for CRAMP-mediated synergistic induction of CD73. (**a**–**e**) Th17 cells were cultured for two more days as indicated in Figure 2d and treated with pharmacological inhibitors as indicated. (**a**) RT-PCR analysis of Nt5e (CD73) in CRAMP-stimulated Th17 cells (*n* = 4). Flow cytometry analysis of CD73 expression on Th17 cells treated with SB431542 (TGF-β receptor inhibitor) (**b**) or SB203580 (p38 inhibitor) (**c**); the bar graph indicates the mean fluorescent intensity (MFI) value of CD73 (*n* = 3). (**d**) RT-PCR analysis of Nt5e (CD73) in SB203580-treated Th17 cells (*n* = 3). (**e**) RT-PCR analysis of Gfi-1 in CRAMP-treated Th17 cells (*n* = 4). (**f**) Flow cytometry analysis of CD73 on retroviral Gfi-1 overexpressed (Thy1.1^+^) Th17 cells; the bar graph indicates the mean fluorescent intensity (MFI) value of CD73 (*n* = 3). * *p* < 0.05, ** *p* < 0.01 *** *p* < 0.001 and n.s—not significant (Student’s t-test or one-way ANOVA with post hoc Tukey test).

TGF-β-mediated p38 activation causes downregulated expression of Gfi-1, a negative transcription factor of *Nt5e* that encodes CD73 in mice [18]. Interestingly, we found that CRAMP-mediated induction of CD73 was restricted on Tregs and Th17 cells that are largely generated by TGF-β. Thus, we hypothesized that CRAMP treatment resulted in the synergistic induction of CD73 at the transcription level along with TGF-β. Indeed, treatment of Th17 cells with CRAMP in the presence of TGF-β and T-cell receptor stimulation significantly induced *Nt5e* (Figure 4a), suggesting that CRAMP regulates CD73 expression at the transcription level. To further explore the signaling pathway responsible for the CRAMP-mediated synergistic induction of CD73, we used selective pharmacological inhibitors. Treatment with the selective TGF-β type I receptor kinase inhibitor SB431542 abrogated CD73 expression, resulting in a level lower than that observed in TGF-β-treated control Th17 cells (Figure 4b). This difference suggested that CRAMP-mediated CD73 induction was dependent on the TGF-β type I receptor signaling pathway. Since the TGF-β-p38-Gfi-1 regulatory axis is critical for determination of *Nt5e* expression on Th17 cells [18], we next examined the role of p38 activity in the process by treating the cells with the selective p38 inhibitor SB203580. Indeed, p38 inhibition suppressed CRAMP-mediated induction at both the protein and mRNA levels, strongly suggesting that CRAMP modulates the p38 signaling pathway to regulate CD73 expression on Th17 cells (Figure 4c,d). We next examined whether p38-mediated Gfi-1 suppression is responsible for CRAMP-mediated CD73 induction. As expected, *Gfi-1* expression was suppressed by CRAMP in Th17 cells (Figure 4e), and overexpression of *Gfi-1* abrogated the CRAMP-mediated synergistic induction of CD73 (Figure 4f). Taken together, these results suggested that p38-mediated *Gfi-1* repression is responsible for the synergistic induction of CD73 by CRAMP with TGF-β on Th17 cells.

## 4. Discussion

Here, we provide the first evidence that the antimicrobial peptide CRAMP is a regulator of CD73 expression on mouse Th17 cells. Since the ectonucleotidase CD73 is the rate-limiting enzyme to produce adenosine, a strong immune suppressor, its regulation might be important in many biological contexts, including in response to inflammation and cancer. In a homeostatic condition, CRAMP is mainly produced in the epithelial cells of barrier tissues such as the skin and intestine. Most of these tissues also comprise Th17 cells, suggesting an interaction with CRAMP to modulate the function of Th17 cells under normal or pathological conditions. Th17 cells are also found in many types of tumors, and their roles have been extensively investigated but remain controversial, with some studies showing antitumor functions and others showing protumor functions [37]. One model to explain this apparent paradoxical role of Th17 cells is the plasticity of these cells with respect to the expression of the transcription factor FoxP3. Indeed, tumor-infiltrated FoxP3+ Th17 cells have been detected in several types of tumors, including breast cancer, melanoma, and colon cancers, and are regarded as immune suppressors [38]. CRAMP expression is also highly upregulated in these tumors, and infiltrated Th17 cells can be stimulated to express FoxP3 and thereby carry out their immune-suppressive roles in the TME. However, we found that CRAMP does not directly influence the expression of FoxP3 on Th17 cells, although it functions together with TGF-β to directly induce CD73 on Th17 cells. Alternatively, the paradoxical roles of Th17 cells in the TME may be related to the alteration of Th17 cells from an antitumor to protumor function via CD73 upregulation. A recent study demonstrated that IL-1β-polarized Th17 cells are effector cells against tumors, whereas TGF-β-polarized Th17 cells do not have such a potent antitumor effect, which was attributed to the induction of CD73 by TGF-β to support tumor growth [18,39]. Importantly, CD73^+^ tumor-infiltrated T cells have been detected in human ovarian and breast cancers [40], implicating a role of CD73 in regulating T cells in the TME. Considering our results and these previous findings, it is plausible that CRAMP present at a high concentration in breast cancer, ovarian cancer, and melanoma serves as a key regulator of CD73 induction on tumor-infiltrated T cells together with TGF-β.

Given the importance of CD73 in many aspects of immunity, its regulation mode has been extensively studied. The CD73 expression level is regulated via transcriptional regulation. In humans and rats, SP1, SMADs, and hypoxia-inducible factor-1 activate transcription via direct binding to the promoter region of *NT5E* [41,42,43], whereas peroxisome proliferator-activated receptor gamma (PPAR-γ) suppresses its transcription in human colorectal cancer [44]. In Th17 cells, Gfi-1 can bind to the upstream region of *Nt5e*, resulting in suppressed expression of *Nt5e* [18]. Interestingly, TGF-β-mediated activation of p38 inhibited *Gfi-1* transcription, suggesting that the TGF-β–p38–*Gfi-1* regulatory axis is critical for the activation of *Nt5e*. In addition to these studies, we found evidence for the transcriptional regulation of CD73 (*Nt5e*) in Th17 and Treg cells via CRAMP in mice. CRAMP is suggested to play a role in activating *Nt5e* in Th17 cells in a TGF-β-dependent manner, since CRAMP itself could not induce CD73 and synergistic induction with TGF-β was completely abrogated by treatment with a pharmacological inhibitor of p38. In addition, CRAMP suppressed the transcriptional activation of *Gfi-1*, and *Gfi-1* overexpression prevented the CRAMP-mediated synergistic induction of CD73. These results suggest that CRAMP modulates the p38 signaling pathway to inhibit *Gfi-1*, thereby upregulating the CD73 expression level on Th17 cells. Identifying the receptors of CRAMP in Th17 cells might provide more insight into the mechanism by which CRAMP regulates the CD73 expression level on Th17 cells.

In addition to CD73 induction, CRAMP was shown to induce apoptosis during the activation of naïve mouse T cells and in differentiated Tregs and Th17 cells. This feature was more prominent with treatment of high concentrations of CRAMP, suggesting that Tregs and Th17 cells expressing CD73 undergo apoptosis in the presence of high levels of CRAMP, similar to the TME. These results are somewhat paradoxical, suggesting that CRAMP might generate suppressive immune cells but also kills these cells in the TME. However, apoptotic Tregs were shown to have a superior suppressive function to living Tregs. Maj et al. [45] demonstrated that apoptotic Tregs release ATP, which is then converted into adenosine by CD39 and CD73. The increase in adenosine, in turn, stimulates the A2a pathway to exert an immune-suppressive effect, supporting immune checkpoint blockade resistance in tumors [45]. This speculation is in line with a previous model showing that immune cell death is a regulatory mode contributing to generation of the immune-suppressive environment of tumors [29,30]. Therefore, it is possible that effector T cell death accompanying enhanced CD73 expression mediated by CRAMP is not a simple consequence of its bactericidal function via cell membrane destruction, but is rather a broader regulatory mechanism to generate an immune suppression environment, particularly in tumors.

Overall, our study extends the roles of CRAMP from an antimicrobial peptide to an immune modulatory peptide via CD73 induction along with apoptosis induction. Since CRAMP also stimulates the proliferation of tumor cells and induces metastasis, it is very plausible that CRAMP secreted from tumors is a potent protumor substance. However, we did not explore this possibility directly in the tumor environment, which is a limitation of our current study. Therefore, further studies are needed to determine whether CRAMP indeed functions as a protumor substance via CD73 upregulation in effector T cells using appropriate tumor-bearing animal models. With this confirmation, our findings along with previous related work can suggest CRAMP as a promising therapeutic candidate for intervention to improve the responsiveness of tumors to immunotherapy.
